# Research progress on the association of maternal high-fat diet with offspring neurodevelopment and susceptibility to Parkinson's disease

**DOI:** 10.3389/fnut.2026.1907443

**Published:** 2026-07-24

**Authors:** Qi Li, Zhi Jia, Musi Ji, Bayaer Nashun

**Affiliations:** 1 School of Basic Medical Sciences, Inner Mongolia Medical University, Hohhot, China; 2 Department of Palliative Care, Hohhot Hospital of Traditional Chinese Medicine and Mongolian Medicine, Hohhot, China

**Keywords:** clinical translation, maternal high-fat diet, NF-κB, offspring neurodevelopment, Parkinson's disease susceptibility, Pink1/Parkin, the marker of early-warning

## Abstract

Maternal high-fat diet (mHFD) is a growing global nutritional concern during pregnancy. It induces maternal systemic low-grade inflammation and oxidative stress, reshaping the intrauterine milieu via the placenta and causing selective developmental impairments in offspring midbrain dopaminergic (DA) neurons—including aberrant precursor proliferation/differentiation, simplified synapses, and nigrostriatal circuit deficits—which may increase adulthood Parkinson's disease (PD) susceptibility. Mechanistically, mHFD acts through two synergistic pathways: persistent activation of the insulin resistant–NF-κB inflammatory axis and suppression of PINK1/Parkin-mediated mitophagy, mutually reinforcing and compromising DA neuron resilience. This article reviews the pathological process of mHFD-mediated remodeling of the intrauterine microenvironment to increase the susceptibility of offspring PD and its two core mechanism pathways: the continuous activation of the IR-NF-κB inflammatory pathway and the functional inhibition of the PINK1/Parkin-mediated mitophagy pathway. On this basis, multi-dimensional early warning markers based on inflammatory factors, mitophagy-related molecules, epigenetic markers and nutritional exposure indicators, as well as potential intervention strategies such as nutritional supplementation, anti-inflammatory and pro-mitophagy targeting the above pathways were summarized, in order to provide a theoretical reference for the primary prevention of PD.

## 1 Introduction

Among the many early life risk factors, maternal high-fat diet (mHFD) has become an increasingly serious nutritional problem during pregnancy worldwide. This dietary pattern usually refers to a diet with a fat-to-energy ratio of more than 35% (1). From the perspective of nutritional epidemiology, the connotation of “high fat” is far more than the increase in energy supply ratio, and its core harm lies in the serious imbalance of dietary fatty acid composition (2). Typical Western high-fat diet is rich in saturated fatty acids (SFA) such as palmitic acid and n-6 polyunsaturated fatty acids (PUFA), while long-chain n-3 PUFA, especially docosahexaenoic acid (DHA), are seriously deficient (3). This high n-6/n-3 ratio state is not only a core nutritional incentive that drives maternal metabolic inflammation, but also directly changes the selective transport spectrum of essential fatty acids in the placenta, transforming a simple “calorie excess” into a far-reaching “nutritional programming signal” (4, 5). Animal experiments and population studies have shown that a high-fat diet can induce excessive fat accumulation and homeostasis disorders, leading to a variety of metabolic disorders (6). With the globalization of Western dietary patterns, the dietary fat intake of women of childbearing age before and during pregnancy continues to rise, which has become a public health issue that cannot be ignored. The core pathological feature of mHFD is the continuous enhancement of maternal systemic low-grade chronic inflammation and oxidative stress (7). This pathological state can reshape the fetal intrauterine microenvironment through the placental barrier, thereby interfering with the central nervous system. In particular, it causes selective damage to the mesencephalic dopaminergic (DAergic) system, which has extremely high energy metabolism requirements and is highly sensitive to inflammation and oxidative damage (7, 8). Studies have shown that maternal metabolic status during pregnancy increases the risk of autism or other neurodevelopmental disorders in their children (9). At present, the global population is accelerating into an aging society. The prevalence of neurodegenerative diseases represented by Parkinson's disease (PD) continues to rise, and the disease burden is increasing. However, there is still a lack of effective early warning and radical treatment. In this context, it is of great scientific significance and public health value to explore the potential programming effect of early life environmental factors on PD susceptibility. PD is the world's second most common disease, and its incidence has continued to rise over the past half century (10). Statistics show that between 1990 and 2015, the number of PD patients worldwide reached more than 6 million; affected by an aging population, this number is expected to double again to more than 12 million by 2040 (11). Previous studies have focused on the role of environmental changes and genetic mutations in PD in adulthood. The Developmental Origins of Health and Disease (DOHaD) hypothesis proposed by existing studies provides a new perspective for the analysis of PD. In 1986, Barker speculated based on historical data that the adverse environment in the early life will permanently affect the body structure and function, thereby increasing the risk of chronic diseases in later life. This argument was originally called the “fetal origin hypothesis” (12). In 2003, the International DOHaD Society was established. Previously, some scholars have pointed out that environmental factors in early life, especially the intrauterine environment, can have a profound impact on the long-term health of individuals. As the direct environment of fetal development, the physiological state of the mother affects the development trajectory of the offspring through complex biological mechanisms (13). The “fetal origin hypothesis” is extended to a more comprehensive “developmental origin” theory, and the influence window is extended to the early stage after birth.

At present, the research on the correlation mechanism between mHFD and the susceptibility of offspring to PD is still in the exploratory stage. The existing animal experiments and clinical cohort studies have preliminarily revealed that insulin resistance-related signaling pathway disorders and mitophagy dysfunction may be the core regulatory links (14). Based on the above background, this paper focuses on the exposure factor of mHFD, reviews its specific damage to the development of DAergic system in the midbrain of offspring, and focuses on the synergistic pathogenic mechanism of IR-NF-κB inflammatory pathway and PINK1/Parkin mitophagy pathway in increasing the susceptibility of offspring to PD. On this basis, this paper will also explore the prospects of the above mechanisms into clinical transformation, including the development of early warning biomarkers and the exploration of targeted intervention strategies, as well as the current core evidence gaps and challenges, so as to provide reference for the improvement of PD etiology theory and the formulation of early prevention and control strategies.

## 2 Maternal high-fat diet induces adverse intrauterine microenvironment and developmental damage of dopaminergic nervous system in offspring

A high-fat diet, that is, a fat-to-energy ratio exceeding 35% of total energy intake, is a common and high-risk metabolic health problem during pregnancy worldwide (1). A large number of clinical and animal studies have confirmed that the above metabolic abnormalities can change the fetal intrauterine environment through the placental barrier, interfere with the proliferation and differentiation of neural stem cells, synapse formation and neurotransmitter homeostasis, and lead to abnormal development of the offspring nervous system (15). Based on the DOHaD hypothesis, this developmental damage is not the end point. When abnormalities are concentrated in the midbrain dopaminergic system, it will reduce the functional reserve of dopaminergic neurons (DAergic neurons) in the adult offspring, thereby increasing the susceptibility of PD. This chapter will elaborate the specific ways of mHFD-induced intrauterine adverse microenvironment and the specific damage of this microenvironment to the development of midbrain DA system, focusing on the core role of inflammation and oxidative stress.

### 2.1 Maternal high-fat diet induces maternal chronic low-grade inflammation and placental barrier dysfunction

Based on the DOHaD theory, pregnancy is a critical window period for fetal neuroplasticity, and intrauterine lipid exposure can permanently program the developmental trajectory of offspring nerve cells. Studies have shown that maternal high-fat diet during pregnancy is closely related to the increase of long-term adverse health risks of offspring, covering cardiovascular diseases, metabolic diseases, immune dysfunction and neurodevelopmental disorders (15, 16). Studies have shown that continuous high-fat intake during pregnancy can break maternal lipid metabolism and immune homeostasis, driving systemic chronic low-grade inflammation and oxidative stress (17, 18). This pathological signal is transmitted to the fetus through the placental barrier and continues to interfere with the whole process of embryonic neural development. The midbrain DA neural precursor cells are highly susceptible to inflammation and lipid toxicity, and are prone to irreversible developmental damage.

Excessive fat intake during pregnancy can cause a large accumulation of white fat in the maternal body, extensive infiltration and continuous activation of adipose interstitial macrophages, and the release of classical pro-inflammatory factors such as interleukin-6 (IL-6) and tumor necrosis factor-α (TNF-α), forming a persistent systemic low-grade inflammatory phenotype (18, 19). At the same time, lipid overload will increase the production of reactive oxygen species (ROS), break the balance between oxidation and anti-oxidation, and further amplify the systemic inflammatory cascade (20). Animal experiments showed that the mRNA and protein expression levels of IL-6 and interleukin-1β (IL-1β) in serum and adipose tissue of high-fat pregnant rats were significantly higher than those in the standard diet control group, and the inflammatory signal ran through the whole pregnancy (21). This change may affect the intrauterine environment of the fetus through the placental barrier, thereby interfering with the development of the fetal nervous system (22). As the core organ of maternal-fetal material exchange and immune isolation, the placenta is highly susceptible to maternal high-fat inflammatory environment damage. The high concentration of pro-inflammatory factors and oxidized lipids in maternal circulation continue to stimulate the activation of placental macrophages, the exchange area of placental labyrinth is reduced, and the barrier permeability is abnormally increased. Pro-inflammatory factors can enter the fetal blood circulation from the mother without restriction (23–25). Under physiological conditions, the placenta can selectively transport polyunsaturated fatty acids essential for neurodevelopment, while high-fat exposure will down-regulate placental lipid transporters and change the fatty acid homeostasis in the fetal brain. Inflammatory factors entering the brain through the blood-brain barrier can activate microglia and astrocytes in the fetal brain in advance, initiate central nervous inflammation in advance, and inhibit the proliferation, migration and differentiation of neural stem cells (21). Animal experiments showed that the expression of IL-6 and TNF-α in placenta of pregnant rats with high-fat diet was significantly increased, the expression of placental barrier tight junction protein was down-regulated, and the ability of transplacental inflammatory transport was enhanced (21).

In addition to the passive transplacental transport of the above inflammatory signals, mHFD also directly deprives the key raw materials for fetal neurodevelopment by actively disrupting the nutrient selective transport function of the placenta. Fatty acid transporters (FATP2, FATP4) and intracellular fatty acid binding protein (FABP) expressed in placental trophoblast cells preferentially accumulate DHA and arachidonic acid (ARA) in fetal circulation under physiological conditions (26). However, the overloaded SFA and n-6 fatty acids in maternal hyperlipidemia will competitively inhibit the transport system, resulting in a significant decrease in the proportion of DHA in the fetal circulation (27). Clinical studies have shown that the placental DHA transport efficiency of obese pregnant women is impaired, and the umbilical cord blood DHA level is reduced (28). DHA supplementation during pregnancy can up-regulate the expression of placental fatty acid transporter 4 and improve placental nutrient transport function (29). Since DHA is the core building block of neuronal membrane phospholipids, its congenital deficiency can directly reduce the fluidity of midbrain DAergic neuron membrane and the signal transduction efficiency of membrane receptors.

In summary, mHFD reshapes the intrauterine adverse microenvironment through two independent pathways: one is to induce persistent systemic inflammation and oxidative stress in the mother and construct a circulating inflammatory signal library; the second is to damage the placental structure, material transport and barrier function, and to amplify the cross-generational transmission of inflammation and lipid toxicity to the fetus. The fetus is in a high inflammation and high oxidative stress intrauterine environment for a long time, and the whole brain nerve development is inhibited. The metabolism of DA neural precursor cells in the ventral midbrain is active and the endogenous antioxidant reserve is weak, which will produce specific developmental defects different from other brain regions.

### 2.2 Intrauterine high-fat inflammatory microenvironment specifically damages the development of dopaminergic system in offspring

During intrauterine development, maternal nutrition, lifestyle, anxiety, stress and other external stimuli can significantly affect fetal development (30). Environmentally harmful factors can induce maternal inflammatory state and change the nutritional supply of the fetus, and have a significant impact on brain morphogenesis and neurological outcomes through epigenetic mechanisms. Therefore, a healthy intrauterine environment is a key factor to support normal fetal brain development (30). Fetal brain intact neurogenesis relies on stabilizing the low-inflammatory intrauterine microenvironment, followed by neural stem cell proliferation, neuronal differentiation, long-distance migration, and synaptic network construction. The high IL-6 and TNF-α intrauterine microenvironment caused by maternal high-fat diet can inhibit the proliferation of neural stem cells and accelerate the apoptosis of immature neurons. The expression of brain-derived neurotrophic factor (BDNF) in the whole brain is significantly down-regulated, and the complexity of the whole brain neural circuit connection is decreased (31–33). Compared with cerebral cortex and hippocampus, DA neural precursor cells that regulate movement in the midbrain are congenitally susceptible to lipid and inflammatory stimulation, which will produce specific developmental damage, which is the core structural premise of adult PD susceptibility in offspring (34). The susceptibility is due to the rigid dependence on n-3 long-chain PUFA, especially DHA. DHA is the most abundant PUFA in the gray matter of the brain, accounting for 30%−40% of phospholipid fatty acids in the gray matter of the cerebral cortex (35). Prenatal n-3 deficiency leads to a decrease in DHA in the substantia nigra of offspring, a simplification of dendritic branches of tyrosine hydroxylase (TH) positive neurons and a decrease in the total number of DA neurons (36). This structural vulnerability makes DAergic neurons enter a metabolically fragile state during the developmental window period, with decreased membrane fluidity and signal transduction efficiency, increased basic oxidative stress, and more sensitive to inflammatory attacks (37). Therefore, the pro-inflammatory signals transmitted by mHFD across the placenta, such as IL-6 and TNF-α, act on the DA system that is already fragile due to insufficient supply of DHA, and the superposition effect of the two is much greater than the effect of any single factor.

TH is a specific marker of mature DAergic neurons. The number of positive cells and the level of protein expression directly reflect the reserve and maturation of DA neurons (38). Animal experiments have shown that mHFD can lead to a decrease in the number of TH -positive neurons in the substantia nigra of the offspring and a decrease in the complexity of synaptic branches of residual neurons (39). The differentiation of DA neurons is blocked, the expression of key transcription factors LMX1 A, NURR1 and PITX3 in midbrain DA development is disordered, and the overall expression level is down-regulated. The embryonic DA neural precursor cell pool is congenitally reduced, resulting in a permanent decrease in the total amount of DA neurons (40–42). The injury originated from the embryonic midbrain differentiation stage, which was a congenital intrauterine programming defect and not caused by postnatal stimulation. The deficiency of congenital DA neuron reserve will weaken the compensatory ability of neurons for a long time. When middle-aged and elderly people suffer from aging and environmental toxin stimulation, they are more likely to cross the threshold of PD pathogenesis, and DA neurons are progressively lost.

After the differentiation of DA neurons, it is necessary to rely on axon guidance molecules to accurately migrate and project to the striatum to construct a complete substantia nigra-striatum motor regulation loop (43). Intrauterine high-fat inflammatory environment can significantly down-regulate the axon attractant molecule Netrin-1, disrupt the migration trajectory of DA neurons and the efficiency of axon extension, resulting in the deviation of neuron localization, the decrease of DA nerve projection density in striatum and the decrease of synaptic branch complexity (39, 44). In some animal models, the total number of DA neurons in the substantia nigra of the offspring did not decrease significantly, but the synaptic structure of the striatum was sparse, the dopamine release sites were reduced, and the synaptic transmission efficiency was impaired, suggesting that the defect of circuit development is a key pathological change independent of neuron loss, and the combination of the two causes the deficiency of congenital motor circuit function (39, 44).

Microglia and astrocytes in fetal brain are the main effector cells of central inflammation. The pro-inflammatory signal transported across the placenta by mHFD can induce the continuous activation of glial cells in the embryonic period, and the glial cells change from the steady-state monitoring type to the M1 pro-inflammatory phenotype, continuously secrete IL-1β and IL-6, and release local inflammation in the brain (45, 46). It can also promote the proliferation of astrocytes by increasing the activity of NF-κB in the substantia nigra and increasing the expression of pro-inflammatory genes such as IL-6, which indirectly leads to changes in synaptic function (47). The expression of Iba1 (microglia marker) and GFAP (astrocyte marker) in the offspring brain tissue of high-fat exposure offspring was significantly increased, accompanied by the continuous down-regulation of BDNF in the brain, which further lost the neurotrophic protective effect and aggravated the vulnerability of DA cells (48).

The effects of the above inflammatory response and oxidative stress on the nervous system of the offspring are multifaceted, especially its damage to the midbrain DAergic system. Therefore, mHFD not only extensively interferes with neural development, but also more specifically impairs the developmental trajectory of mesencephalic DAergic neurons, providing an important structural basis for increased susceptibility to PD in adulthood.

## 3 The potential mechanism by which maternal high-fat diet increase the susceptibility of offspring to PD

At present, the complete life cycle human epidemiological evidence that “mHFD to the increased incidence of PD in adulthood” is still limited, mainly due to the long latency of PD, many confounding factors and the difficulty in establishing causality in retrospective studies. This article focuses on the possible molecular hypothesis based on the DOHaD framework and animal models, and provides objective verification for the future design of specialized prospective birth cohorts. This chapter first describes how fetal DAergic neurodevelopmental abnormalities constitute the structural and functional basis of PD, and then focuses on the synergistic effect of two core pathways-insulin resistance-persistent activation of NF-κB pathway and PINK1/Parkin-mediated mitophagy pathway inhibition-in the susceptibility of mHFD to programmed offspring PD.

### 3.1 Fetal dopaminergic neurodevelopmental abnormalities: the structural basis of PD susceptibility

The core pathological feature of PD is the loss of DAergic neurons in the substantia nigra of the midbrain, and the pro-inflammatory immune-mediated mechanism plays a key role in the occurrence and progression of the disease (49). From the perspective of developmental origin, the vulnerability of DA neurons in adulthood is not only induced by acquired stimulation. Innate programming damage during the differentiation and loop construction of DA neural precursor cells in embryonic stage will permanently compress the functional reserve of neurons and reduce the ability of individuals to tolerate damage from the root (50). These neurons are differentiated from specific precursor cells in the ventral side of the embryonic midbrain and are precisely regulated by key transcription factors such as LMX1A, NURR1, and PITX3 (51). The inflammatory signals transmitted by mHFD through the placenta can directly interfere with the expression timing or level of the above transcription factors, which will lead to the reduction of the size of the midbrain DA neural progenitor cell pool and the disorder of differentiation, and finally the total number of DA neurons is lower than the normal level (39). In addition, newborn DAergic neurons need to precisely migrate to the substantia nigra pars compacta and project axons to the striatum (52). Abnormal migration path or axon guidance signal can lead to neuronal localization error or neural circuit connection error (53). Animal studies have shown that maternal offspring exposed to HFD, the projection of DAergic neurons in the SN reticular part and NAc is impaired, and DA-related behavioral changes occur, but the number of DAergic neurons does not change significantly (54). Intrauterine high-fat environment can change the expression of axon guidance molecules Netrin-1 and Semaphorin or the interference of maternal inflammatory factors on the migration path, resulting in some neuronal localization errors or insufficient striatum innervation (55). Although this structural defect of the substantia nigra-striatum circuit itself is not sufficient to immediately lead to PD, it significantly reduces the DAergic system reserve of adult individuals. In the face of age-related mitochondrial dysfunction, environmental toxin exposure or α-syn aggregation, the compensatory ability of DAergic neurons is worse and it is easier to cross the clinical threshold of PD. Therefore, the abnormal development of DAergic neurons in the fetus is a structural prerequisite for maternal metabolic abnormalities to increase the susceptibility of offspring to PD. However, the above developmental damage does not occur in isolation, but is likely to be a concrete manifestation of the dysfunction of the two core pathways described below during development.

### 3.2 Dual signaling pathways synergistically cause disease: from developmental defects to adult vulnerability

In addition to the above-mentioned developmental outcomes, mHFD can also establish a long-standing pathological susceptibility state in the offspring's brain by continuously activating or inhibiting specific signaling pathways. The dysfunction of these two pathways is not only a direct driver of the developmental defects, but also a molecular basis for adult DAergic neurons to be more sensitive to damage. The existing evidence points to two closely related pathways: insulin resistance-driven NF-κB chronic inflammation pathway and PINK1/Parkin-regulated mitophagy pathway. Both of them synergistically reduce the anti-injury ability of DAergic neurons from the two dimensions of inflammatory imbalance caused by energy metabolism and impaired mitochondrial clearance.

#### 3.2.1 Long-term activation of insulin resistant-NF-κB pathway

**Figure 1:**
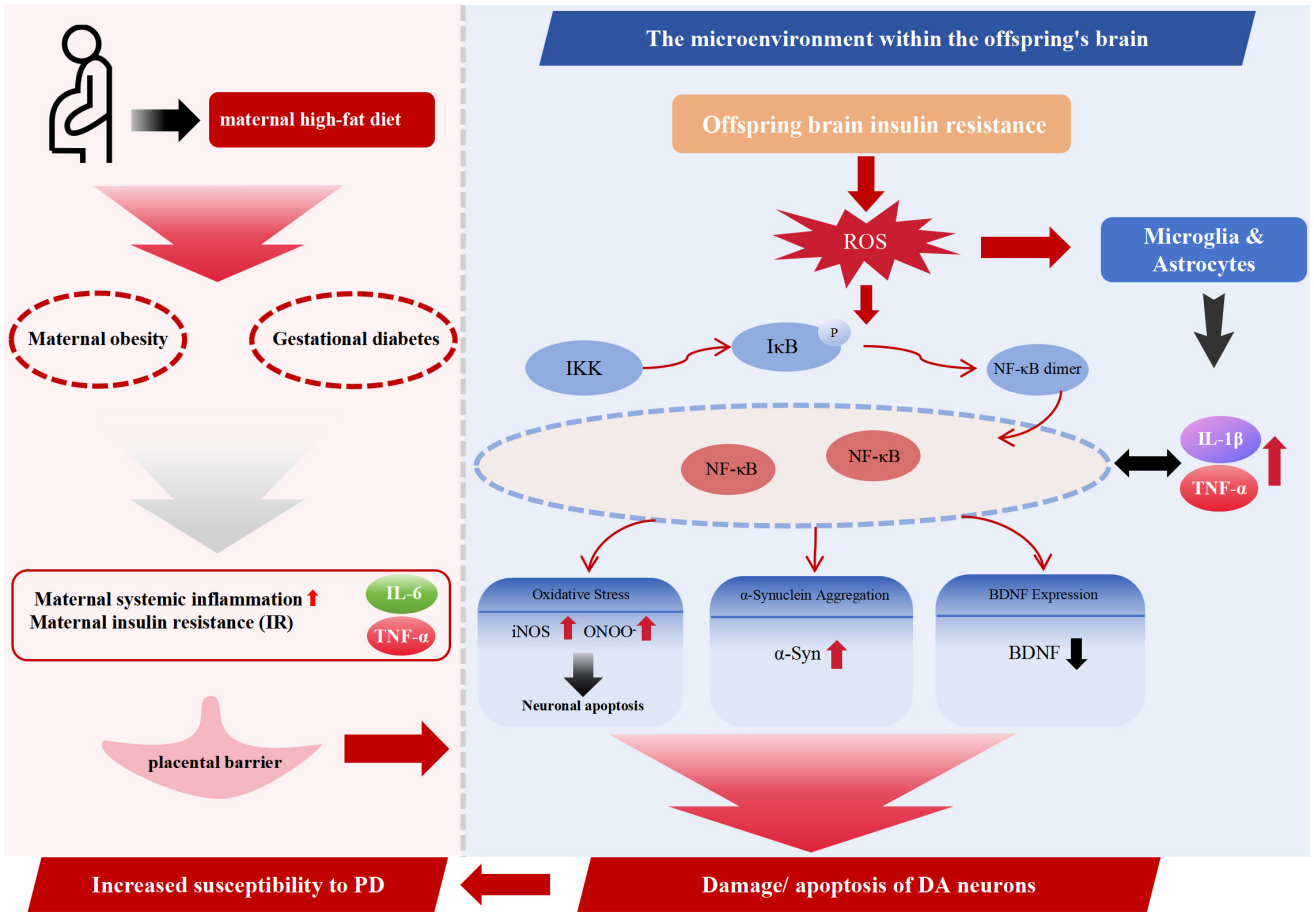
A schematic diagram of the mechanism of mHFD increasing the susceptibility of offspring to PD through the IR-NF-κB pathway. The **left** area showed that mHFD caused systemic inflammation and IR, which were transmitted to the offspring through the placenta. IR in the **right** area of the offspring brain leads to an increase in mitochondrial ROS, activation of the IKK/IκB/NF-κB cascade, up-regulation of pro-inflammatory factors and iNOS, and down-regulation of BDNF. The above changes together cause oxidative nitration damage, energy metabolism disorder and neuronal survival ability decrease, and aggravate DAergic neuron damage. The red arrow indicates the change of enhancement or damage in the abnormal state of mHFD.

mHFD induces maternal systemic lipid accumulation and peripheral insulin resistance (IR), this IR state will affect the offspring through the placental barrier for a long time, resulting in the offspring being prone to IR after birth, which in turn increases the susceptibility of offspring PD by activating the NF-κB signaling pathway (56, 57) (Figure 1) . In the nervous system, insulin signaling not only regulates glucose uptake, but also affects energy metabolism, synaptic plasticity, and dopamine turnover in DAergic neurons (58). When IR occurs in the brain of the offspring, on the one hand, it will lead to insufficient energy supply of neurons and metabolic dysfunction, which in turn affects the survival and function of neurons. More importantly, IR will continue to activate NF-κB through a chronic inflammatory cascade driven by metabolic abnormalities (59, 60).

NF-κB is an important transcription factor, which plays a key role in inflammatory response, immune response and apoptosis. Recent studies have found that NF-κB plays an important role in nervous system diseases such as epilepsy, stroke, Alzheimer's disease, PD and tumorigenesis (61). IR activates the NF-κB signaling pathway through the interaction of metabolism and inflammation, so that it is over-activated for a long time. On the one hand, IR causes an increase in ROS production in neurons. ROS activates the IκB kinase complex, promotes the phosphorylation and degradation of IκB, and releases NF-κB into the nucleus to initiate pro-inflammatory gene transcription (62). On the other hand, intrauterine high-fat inflammation can activate microglia and astrocytes in the fetal brain in advance. Activated glial cells continuously secrete pro-inflammatory factors such as IL-1β and TNF-α, and further amplify the signal intensity of NF-κB through paracrine circulation to form a persistent chronic neuroinflammatory microenvironment (47). It should be noted that the above mechanisms are mainly derived from the analysis of offspring brain tissue in high-fat diet or GDM animal models, as well as *in vitro* neuron-glial co-culture experiments, and human autopsy or brain imaging evidence is still accumulating.

Long-term activation of NF-κB signaling pathway can damage DAergic neurons through multiple pathways. The up-regulation of iNOS expression can increase the production of nitric oxide (NO). NO combines with superoxide anion (O^2−^) to form hydrogen peroxide nitrite (ONOO^−^), which promotes the production of neurotoxic substances, causes oxidative nitration damage of mitochondria and DNA, and accelerates neuronal apoptosis (63). The second is the abnormal aggregation of α-syn. In the MPTP model, NF-κB inhibitor can reduce the activity of SNCA promoter and accompanied by the decrease of α-syn protein, and the aggregation of α-syn to form Lewy bodies is a typical pathological feature of PD (64). Finally, NF-κB activation can down-regulate the expression of BDNF, which is a key factor in maintaining the survival and function of DAergic neurons. The lack of BDNF will further reduce the anti-injury ability of neurons (65). Therefore, the long-term activation of the IR-NF-κB axis in the offspring brain induced by mHFD constitutes a vicious cycle of “metabolic disorders-chronic neuroinflammation-DAergic neuronal damage” (Figure 1). However, this vicious cycle is not only indirectly regulated by the insulin signal, but also the specific fatty acid molecule itself can directly act on the key nodes of the pathway. SFA can directly activate IKK-β through the TLR4/MyD88 pathway and initiate NF-κB nuclear translocation independent of insulin signaling (66). On the other hand, n-3 PUFA can inhibit IKK-β/NF-κB signaling through GPR120 receptor activation (67). This means that the activation of NF-κB pathway by mHFD is not only an indirect consequence of metabolic disorders, but also a direct molecular effect of dietary fatty acid types.

#### 3.2.2 Inhibition of mitophagy-PINK1/Parkin pathway

Mitochondria can maintain the electrophysiological activity of DAergic neurons, the synthesis and transport of neurotransmitters, and the integrity of cell structure. It is also the main site of ROS production. The maintenance of its functional homeostasis is highly dependent on the mitophagy process (68). Impaired mitochondrial homeostasis, altered mitochondrial morphology and transport are hallmarks of PD and PD-specific patients (69). DAergic neurons have the characteristics of high energy consumption and high autonomous electrical activity, and their functional integrity is highly dependent on mitochondrial quality control. Mitochondrial quality control is a regulatory network that maintains the number, morphology and function of mitochondria through multiple mechanisms such as fusion/division dynamic balance, selective autophagy clearance of damaged mitochondria and mitochondrial biogenesis (70). Mitochondrial autophagy mediated by PINK1/Parkin is the core mechanism of damaged mitochondrial clearance (71).

mHFD have long-term inhibition of PINK1/Parkin pathway activity in the offspring brain in two main ways: First, epigenetic inhibition of gene expression. mHFD can change the epigenetic modification profile of offspring brain tissue. Animal experiments have shown that mHFD can cause histone deacetylation modification of Parkin locus and inhibit its transcriptional expression (72). In addition, mHFD can interfere with carbon metabolism, which is the core metabolic pathway of methyl donors, and its disorder can widely change the DNA methylation profile (73). Although the direct evidence of PINK1 promoter methylation in the high-fat diet model has yet to be accumulated, studies have confirmed that abnormal maternal nutritional status can change the DNA methylation level of PINK1 gene in fetal tissues (74). Given that epigenetic modifications are tissue-specific, whether such changes also occur at the PINK1 locus in the midbrain needs further study. Second, oxidative stress destroys protein function. High-fat diet induced the increase of maternal systemic oxidative stress. ROS and lipid peroxides entered the fetal brain tissue through the placenta, so that the basic ROS level in the fetal brain continued to rise, and the activity of antioxidant enzymes in the brain tissue was simultaneously down-regulated (75). Elevated ROS can oxidize the cysteine at the active site of Parkin, resulting in the loss of its E3 ubiquitin ligase activity. At the same time, the high-fat lipid environment can inhibit the transcription efficiency of PINK1 gene and reduce the phosphorylation level of PINK1 protein. Even if the mitochondria have membrane potential damage, Parkin cannot be recruited normally to initiate the mitophagy process, and the damaged mitochondria cannot be cleared in time (76, 77).

**Figure 2:**
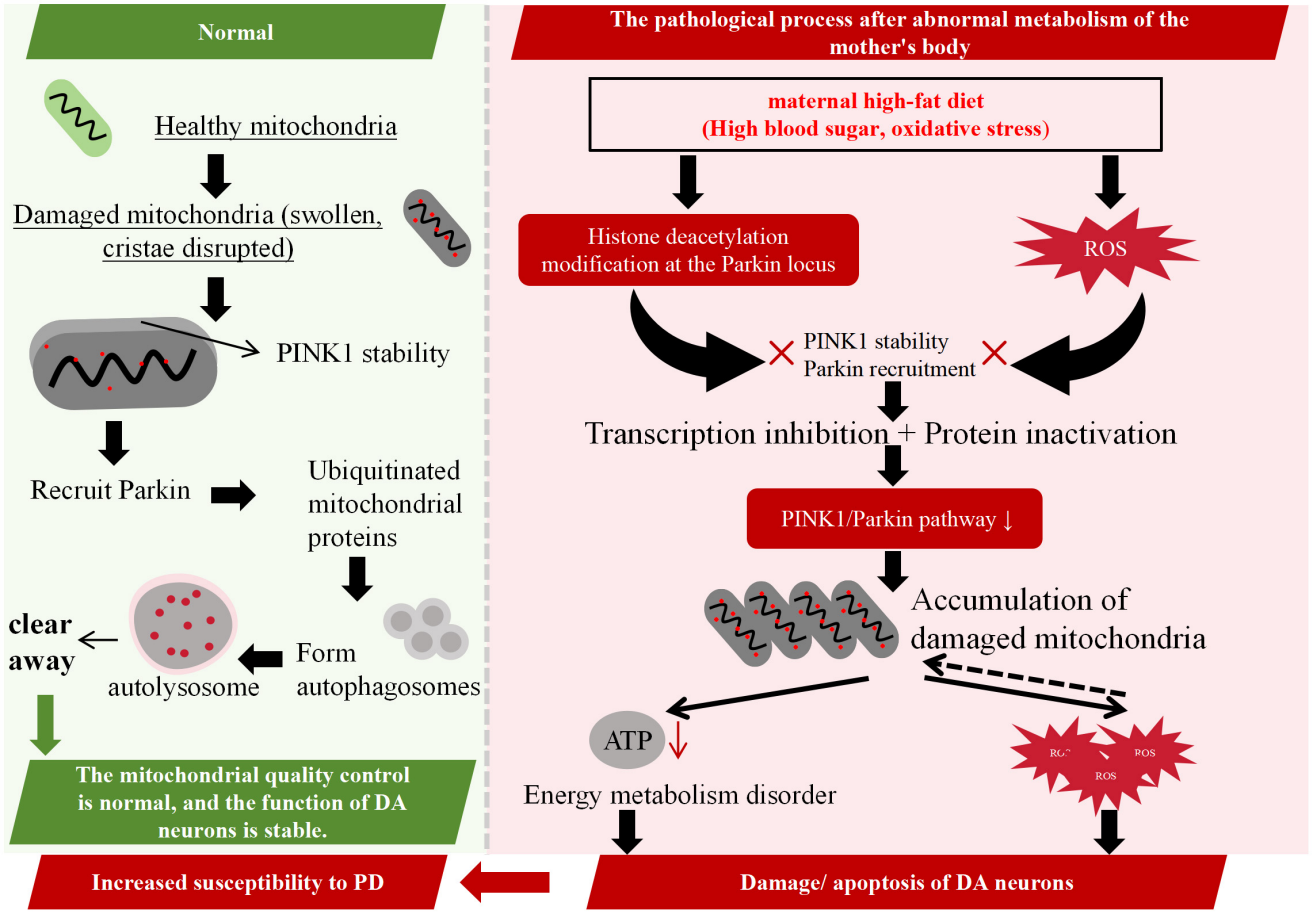
A schematic diagram of the mechanism of mHFD increasing the susceptibility of offspring PD by inhibiting PINK1/Parkin-mediated mitophagy. Under normal conditions **(left)**, PINK1/Parkin pathway maintains mitochondrial quality control by removing damaged mitochondria; mHFD **(right)** lead to Parkin inactivation by inhibiting gene transcription and oxidative stress, double blocking the pathway. The accumulation of damaged mitochondria leads to energy crisis and excessive production of ROS, which eventually leads to the degeneration of DAergic neurons. Red X indicates inhibition or blocking. The green background represents the physiological process and the red background represents the pathological change.

The direct consequence of functional inhibition of PINK1/Parkin pathway is that damaged mitochondria cannot be selectively removed and accumulate in DAergic neurons (78). On the one hand, the damaged mitochondria can not effectively produce ATP, leading to neuronal energy metabolism disorders, unable to maintain normal electrical activity and neurotransmitter synthesis (79). On the other hand, the abnormal function of the electron transport chain of the damaged mitochondria will produce more ROS, forming a “vicious cycle of oxidative stress-mitochondrial damage.” ROS can oxidize dopamine to produce neurotoxins, damage DNA and proteins, and ultimately induce apoptosis of DAergic neurons (80). In conclusion, the PINK1/Parkin pathway is the core of regulating mitophagy. If its function is inhibited by maternal metabolic abnormalities for a long time, it will lead to the accumulation of damaged mitochondria in offspring, lead to energy metabolism disorders, excessive production of ROS, and ultimately increase the susceptibility of DAergic neurons to damage and PD. The functional inhibition of this pathway is a key molecular bridge connecting early metabolic abnormalities and late mitochondrial dysfunction (Figure 2). In addition, the efficiency of mitophagy is also directly regulated by nutrient availability. DHA is a key component of cardiolipin and is indispensable for maintaining the integrity of the mitochondrial inner membrane (81). When DHA is insufficient, the stability of mitochondrial membrane potential decreases, and the accumulation efficiency of PINK1 in the outer membrane of mitochondria decreases. Even if Parkin function is normal, the clearance signal of damaged mitochondria is also weakened due to “substrate recognition disorder” (82). Therefore, the inhibition of PINK1/Parkin pathway by mHFD also superimposes the nutritional factor of mitochondrial membrane structure fragility caused by insufficient DHA supply.

#### 3.2.3 Synergistic effects of the two pathways

The IR-NF-κB pathway and the PINK1/Parkin pathway are not independent, but mutually reinforcing. The upstream IR-NF-κB inflammatory axis is dominant, and its activation directly inhibits Parkin transcription and induces its oxidative inactivation, thereby downstream blocking PINK1/Parkin-mediated mitophagy; autophagy disorder leads to the accumulation of damaged mitochondria and the outbreak of ROS, which in turn strongly activates NF-κB and forms a closed-loop amplification (83, 84). Therefore, mHFD may simultaneously damage two pathways, allowing DAergic neurons to simultaneously withstand the dual effects of chronic inflammatory attacks and intrinsic repair defects. At present, this synergistic hypothesis is mainly supported by a variety of PD animal models and phosphoproteomic analysis of brain tissue in offspring of mHFD, which needs to be further verified in conditional gene knockout or *in vivo* dual-pathway intervention models.

In summary, mHFD, through the cross-effect of two molecular pathways, buried two mutually amplified pathological tendencies in the offspring's nervous system-proinflammatory environment and mitochondrial quality control failure. These changes, together with fetal DAergic neurodevelopmental defects, constitute the biological basis of increased susceptibility to PD in adulthood.

## 4 Clinical transformation value and existing challenges

### 4.1 Early warning biomarkers based on core pathways

The clinical diagnosis of PD mainly depends on motor symptoms such as bradykinesia, myotonia, and resting tremor, but the loss of DA neurons in the substantia nigra has exceeded 50%−60%. If high-risk individuals can be identified decades before the onset of symptoms, it will win a valuable time window for early intervention (Table 1). Based on the two core pathways discussed above, the following four types of biomarkers have potential early warning value. In the future, it is expected to improve the risk prediction efficiency through multi-index joint models such as PINK1 combined with inflammatory factors and clinical precursor symptom evaluation.

**Table 1:** Comparison of PD early warning biomarkers based on two core pathways.

Marker category	Representative indexes	Detected samples	Time point of warning	Dominance	limitation	Research stage
Inflammatory markers (85, 86)	NLR, IL-6, TNF-α	Peripheral blood	Children/adolescence	The detection is convenient and low cost	It has low specificity and is interfered by other inflammatory states	It has been used in prospective validation of PD patients and high-risk groups
Mitophagy-related molecules (87, 88)	Plasma PINK1, PARKIN, PGAM5, p-Ser65-Ub	Peripheral blood	Children/adolescence	The mechanism is highly specific and directly related to the core pathway	The threshold of detection technology is high	It has been used in prospective validation of PD patients and high-risk groups
Epigenetic markers	DNA methylation in PINK1/Parkin promoter region	Peripheral blood wbc	At birth	Early warning time is the earliest and can be screened at birth	Tissue-specific issues requiring large-scale validation	In the animal model stage, clinical transformation still needs to be verified by birth cohort
Metabolic syndrome related indicators (90–92)	Maternal BMI during pregnancy, weight gain during pregnancy, GDM; waist circumference, HOMA-IR, TG /HDL-C of offspring	Routine physical examination/prenatal examination	Prenatal and offspring from childhood	Early warning time is the earliest and can be screened at birth	Low specificity, interference by multiple factors	It has been used for high-risk identification, and cohort validation of PD association is required
Nutritional exposure specific markers (93, 94)	Maternal erythrocytemembrane DHA content, plasma n-6/n-3 ratio, umbilical cord blood phospholipid fatty acid profile during pregnancy	Peripheral blood/umbilical cord blood	Pregnancy/birth	Direct quantification of fetal PUFA exposure intensity to achieve “exposure assessment”	It is necessary to combine mechanism markers to reflect nutritional exposure rather than direct injury	In the epidemiological association stage, prospective cohort validation is required

NLR is the ratio of neutrophils to lymphocytes; iL-6 is interleukin-6; tNF-α is tumor necrosis factor-α; pINK1 is PTEN-induced kinase 1; pARKIN is an E3 ubiquitin ligase; pGAM5 is a member of phosphoglycerate mutase family 5; p-Ser65-Ub is phosphorylated Ser65-ubiquitin; bMI is body mass index; gDM is gestational diabetes mellitus; hOMA-IR was used as a steady-state model to evaluate insulin resistance index. TG / HDL-C is the ratio of triglyceride / high density lipoprotein cholesterol. “Used in PD patients” means that the difference of this marker between PD patients and healthy controls has been verified by clinical studies. “PD diagnosis research stage” means that its value in PD diagnosis is being studied. The “animal model stage” means that the evidence is only obtained in animal experiments and remains to be clinically transformed.

#### 4.1.1 Inflammatory markers

mHFD established a chronic pro-inflammatory state in the offspring brain through the IR-NF-κB pathway. In peripheral blood, neutrophil-to-lymphocyte ratio (NLR) has been reported to be significantly higher in PD patients than in healthy controls, and can be used as a simple indicator of systemic inflammation (85). In addition, pro-inflammatory factors such as IL-6 and TNF-α also showed an increasing trend in peripheral blood of PD patients (86). For high-risk offspring with a history of mHFD exposure, the above inflammatory indicators can be regularly detected in childhood or adolescence to dynamically assess the risk of neuroinflammation.

#### 4.1.2 Mitophagy-related molecules

PINK1/Parkin pathway is the core of mitochondrial quality control. Recent clinical studies have shown that plasma PINK1 levels in patients with idiopathic PD are significantly higher than those in healthy controls, with an area under the receiver operating characteristic curve (AUC) of 0.771, suggesting that plasma PINK1 has the potential to distinguish PD patients from healthy controls (87). Phosphorylated Ser65-ubiquitin has also been reported as a PD-related mitophagy regulatory protein marker (88). In addition, the expression profile of mitophagy-related protein combinations (PINK1, PARKIN, PGAM5) in plasma may be a potential biomarker for PD diagnosis (89). Although the above markers are currently mainly used for the auxiliary diagnosis of patients, their dynamic changes in pre-symptomatic high-risk populations deserve to be verified by prospective cohort studies.

#### 4.1.3 Epigenetic markers

mHFD can permanently change the expression profile of DA neuron-related genes in offspring through epigenetic mechanisms such as DNA methylation and histone modification. Given that epigenetic modifications can be detected in peripheral blood leukocytes, and some changes are established at birth, the methylation level of specific loci is expected to be a PD risk imprint that can be identified at birth. At present, the research in this direction mainly focuses on the animal model stage, and the clinical transformation still needs the support of epigenomic association analysis of large-scale birth cohorts.

#### 4.1.4 Metabolic syndrome related indicators

mHFD directly leads to maternal obesity, insulin resistance, dyslipidemia and other metabolic syndrome components. Pre-pregnancy BMI, weight gain during pregnancy and gestational diabetes mellitus can be used as quantifiable alternative indicators of intrauterine exposure intensity of offspring, which is suitable for early screening (90). The increase of waist circumference (91), the increase of insulin resistance index (92) and the abnormal ratio of triglyceride to high-density lipoprotein cholesterol (91) in the offspring during childhood reflect the continuous metabolic trajectory of intrauterine programming, which is consistent with the functional changes of the two core pathways in time. Although the specificity is low, the combination of inflammatory markers or mitophagy molecules can significantly improve the risk stratification efficiency. It is recommended that high-risk offspring monitor the above metabolic indicators every 2–3 years from puberty.

#### 4.1.5 Nutritional exposure specific markers

In addition to the above mechanism markers, nutritional markers that reflect the level of dietary fatty acid exposure during pregnancy also have early warning value. DHA content in maternal erythrocyte membrane during pregnancy, plasma n-6/n-3 ratio, and umbilical cord blood phospholipid fatty acid profile can directly quantify the PUFA exposure intensity during fetal period. Epidemiological studies have shown that umbilical cord blood DHA levels are positively correlated with cognitive function in children, and high n-6/n-3 ratios are associated with elevated inflammatory markers and increased risk of neurobehavioral problems (93, 94). The combination of these nutritional exposure markers with the aforementioned mechanism markers (inflammatory factors, mitophagy molecules) is expected to construct a complete risk prediction chain from “exposure assessment” to “damage identification.”

### 4.2 Early intervention strategies targeting two pathways

**Figure 3:**
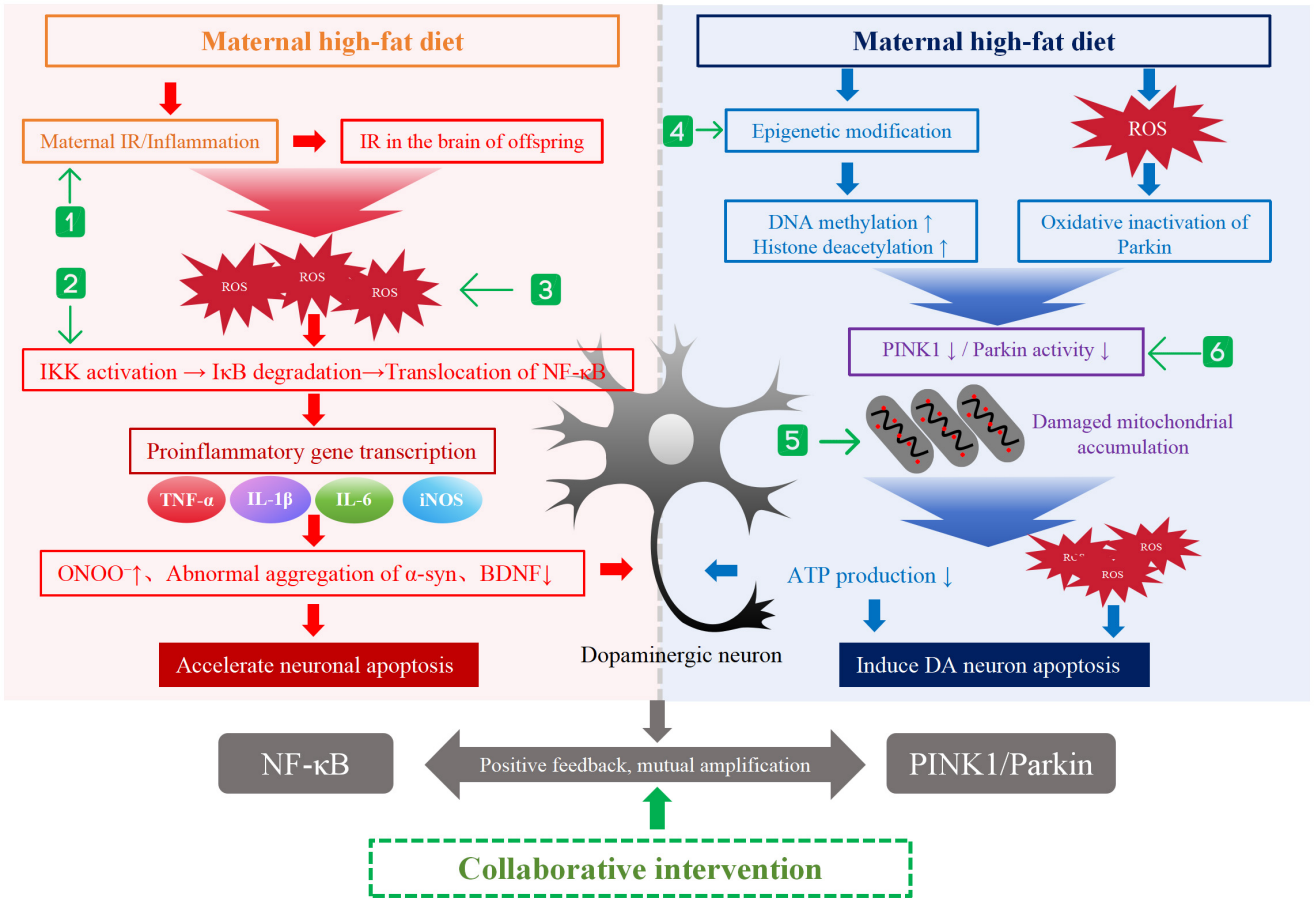
Schematic diagram of clinical transformation targets of two core pathways. The **left** side (red line) is the IR-NF-κB inflammatory pathway, the **right** side (blue line) is the PINK1/Parkin mitophagy pathway, and the DA neurons are in the center. Red and blue solid arrows indicate the direction of pathological promotion; the green arrow indicates the direction of intervention; green numbers 1–6 indicate intervention targets: 1 n-3 PUFA supplementation, 2 IKK-β inhibitors, 3 antioxidants, 4 methyl donor nutrition, 5 mitophagy inducer, 6 nano-targeted delivery system. “↑” indicates enhancement/up-regulation, and “↓” indicates weakening/down-regulation.

Based on the molecular mechanisms of IR-NF-κB pathway and PINK1 / Parkin pathway, a variety of intervention strategies have shown potential protective effects in preclinical or clinical studies. Focusing on DA neurons, the key intervention nodes of the two pathways are shown in Figure 3.

#### 4.2.1 Nutritional optimization during pregnancy: n-3 polyunsaturated fatty acid supplementation

N-3 polyunsaturated fatty acids have clear anti-inflammatory and neuroprotective effects (95). Animal experiments showed that fish oil supplementation during pregnancy could significantly alleviate the adverse effects of high-fat diet on the number of dendritic spines in the cerebral cortex of offspring, reverse the abnormal motor and auditory reflexes caused by high-fat diet, and up-regulate the transcription level of BDNF in male offspring (96). In PD animal models, maternal n-3 PUFA supplementation can improve inflammation-induced neurotoxicity of the DAergic system (97). These findings suggest that n-3 PUFA nutritional intervention in mHFD-exposed mothers may be a safe and feasible neuroprotection strategy for offspring. At present, this strategy has been verified in some preclinical models, but there is still a lack of randomized controlled trials for long-term neurodevelopmental outcomes of human offspring.

#### 4.2.2 Anti-inflammatory and antioxidant intervention

The NF-κB pathway is the core regulatory node of neuroinflammation, and its upstream kinase IKK-β has been regarded as a potential therapeutic target for PD. Natural products such as cardamonin can improve neuroinflammation in PD models by regulating NF-κB signaling; cycloastragenol can target Fpr2 to inhibit TLR4/NF-κB pathway, improve behavioral indicators and enhance neuronal viability in PD mice (98, 99). In addition, resveratrol supplementation has been reported to reduce maternal high-fat diet-induced cognitive and molecular changes in offspring, and its mechanism involves epigenetic regulation (100). At present, most of the above interventions are in the preclinical stage, and the transformation to pregnancy or early life application still needs to systematically evaluate its safety.

#### 4.2.3 Pro-mitophagy strategy

In view of the functional inhibition of PINK1/Parkin pathway, restoring mitophagy activity is another important direction. Recent studies have developed a nanogel system based on dextran/fibronectin, which can achieve brain-targeted mitophagy inducer delivery, inhibit NF-κB signaling, scavenge ROS and promote microglia to transform into anti-inflammatory phenotype in PD models (101). Although this technology is currently designed for patients with PD, its core idea—restoring PINK1/Parkin pathway activity and removing damaged mitochondria through drugs or nutrients can also provide target enlightenment for early intervention of high-risk offspring.

The above intervention strategies target different links of the two pathways, and their action levels, research stages and transformation prospects are different, summarized as shown in Table 2.

**Table 2:** Summary of early intervention strategies targeting two core pathways.

Intervention strategy	Targeting pathway	Link of action	Timing of intervention	Effect evidence	Research stage	Security assessment
n-3PUFA supplement (96, 97)	IR-NF-κB	Anti-inflammatory, antioxidant	Duration of pregnancy	Improve the number of dendritic spines and up-regulation of BDNF expression in offspring (animal experiment)	Preclinical model validation	High safety, can be applied during pregnancy
Cardamonin, cycloastragenol (98, 99)	IR-NF-κB	IKK-b inhibits and blocks the nuclear translocation of NF-κB	Adulthood (current)	Improve neuroinflammation and behavioral indicators of PD model	Preclinical stage	Systematic evaluation of reproductive and developmental toxicity is needed
Resveratrol (100)	Epigenetic + anti-inflammatory	NF-κB inhibition	Duration of pregnancy	Alleviate mHFD diet-induced cognitive changes in offspring (animal experiments)	Preclinical stage	The safety of pregnancy is not clear
Mitochondrial autophagy inducer (101)	PINK1/Parkin	Remove damaged mitochondria	Adulthood (current)	Inhibition of NF-κB, removal of ROS, and promotion of microglial transformation (animal experiments)	Nano-delivery system research and development	Brain targeted delivery technology has not been perfected yet

n-3 PUFA is n-3 polyunsaturated fatty acids; pD is Parkinson's disease; rOS is reactive oxygen species; iKK-β is an IκB kinase-β; nF-κB is nuclear factor-κB. “Preclinical model validation” refers to the validation in animal models; “Preclinical stage” means that it is still in cell or animal experiments and has not yet entered clinical trials. The “nano-delivery system research and development” means that the delivery technology is still in the development stage. “Pregnancy” refers to the intervention of the mother during pregnancy; “Adult” means that the strategy is currently designed only for adult PD patients who have already developed the disease.

### 4.3 The existing core challenges

Although great progress has been made in mechanism research, the transformation from laboratory to clinical still faces multiple obstacles. First, there is a serious lack of direct human prospective cohort evidence. The incubation period of PD is as long as 40–60 years, which makes it extremely difficult to follow up the whole life cycle of PD with the end point of “mHFD exposure to the incidence of PD in adult offspring.” In addition, the historical changes of diagnostic criteria and the interference of a large number of confounding factors, the direct verification of causality has not been achieved so far.

Second, the convertibility of animal models to humans is questionable. There are significant differences in the developmental timing, life span and environmental exposure spectrum of mesencephalic DA neurons between rodents and humans. Whether the pathway changes observed in animal models can occur in the same way in humans needs to be verified by human iPSC-derived DA neurons, brain organoids and autopsy tissue studies.

Third, the intervention window and measures lack certainty. The optimal timing (pre-pregnancy, early pregnancy, late pregnancy or lactation), optimal dose and formula for nutritional optimization during pregnancy are not yet clear; there is a lack of evidence-based clinical guidelines on when to start monitoring, how often to follow up, and when to initiate intervention in high-risk offspring. Fourth, the practical dilemma of ethics and implementation. Long-term drug intervention trials in high-risk offspring face a difficult trade-off between “potential risk” and “intervention benefit,” as well as thorny issues such as the maintenance of subjects' compliance during decades of follow-up, and the effectiveness of interventions without clear endpoints. Overcoming the above challenges one by one will be the key to the field from mechanism cognition to clinical landing.

## 5 Conclusion and prospect

### 5.1 Conclusion

mHFD is an important environmental risk factor for the developmental damage of DA system in the midbrain of offspring. Existing animal experiments and indirect clinical evidence suggest that such abnormalities interfere with the proliferation and differentiation of neural stem cells, synaptic formation, and neurotransmitter homeostasis through the placental barrier, mainly through chronic inflammation, oxidative stress, and epigenetic modification, especially causing structural and functional damage to midbrain DAergic neurons, thereby increasing the susceptibility of offspring to PD in adulthood.

At the molecular mechanism level, The continuous activation of the IR-NF-κB inflammatory pathway and the functional inhibition of the PINK1/Parkin-mediated mitophagy pathway constitute two synergistically amplified pathogenic pathways. The former destroys neuronal energy homeostasis and promotes α-syn aggregation through a vicious cycle of metabolism-inflammation. The latter causes self-enhancement of oxidative stress by impaired mitochondrial clearance disorder, which together reduce the anti-injury ability of DA neurons. The superposition of the above developmental defects and molecular pathological tendencies constitutes the biological basis of increased susceptibility to PD in adulthood.

From the perspective of clinical significance, The above mechanism discovery provides a potential entry point for the early prevention and control of PD: on the one hand, inflammatory factors, mitophagy-related molecules and epigenetic markers based on two pathways are expected to be used for early identification of high-risk offspring; on the other hand, intervention strategies such as n-3 PUFA supplementation, anti-inflammatory and pro-mitophagy have shown potential protective effects. However, there are still significant obstacles to the transformation from mechanism cognition to clinical application, including the lack of prospective cohort evidence, the limitations of animal model extrapolation, and the lack of standardization of intervention strategies. In general, this review supports the applicability of the DOHaD framework in the etiology of PD, suggesting that the early life nutritional environment may be an intervention window that has not been fully valued in the prevention and control of PD.

In summary, this paper not only enriches the etiology theory of PD, but also highlights the importance of early life intervention in the prevention and control of PD. It is suggested that strengthening the metabolic health management of women of childbearing age and preventing and controlling metabolic abnormalities during pregnancy can help reduce the risk of neurodevelopment and the incidence of PD in offspring.

### 5.2 Prospect

In summary, this paper suggests that in the future, we can focus on the following three directions to carry out systematic exploration to promote the gradual deepening of PD research and prevention.

First, construct a prospective birth cohort. To construct a special disease birth cohort of nutrition during pregnancy and long-term neurological health of offspring. Based on the existing maternal and child health care network, it is recommended to include large-scale pregnant women and systematically collect dietary data before and during pregnancy, especially fat composition and fatty acid profile, to conduct long-term tracking of offspring from birth to adulthood, and to provide key human evidence for establishing a direct causal link between mHFD and PD risk. Secondly, By using multi-omics technology, inflammatory factors, mitophagy-related molecules and epigenetic markers were dynamically detected at different developmental stages of the cohort offspring, and combined with motor and non-motor function assessment, a PD risk stratification model that can be used in clinical practice was constructed. Third, Carry out clinical research on early intervention for high-risk groups. The high-risk offspring with mHFD exposure were identified in the cohort, and the safety and preliminary efficacy of nutrition optimization (such as n-3 PUFA supplementation), lifestyle intervention and targeted pathway regulation measures were systematically evaluated to provide evidence-based basis for the development of primary prevention strategies for PD. Through the above multi-dimensional and cross-stage collaborative research, it is expected to make substantial breakthroughs in early warning, targeted intervention and root prevention of PD, and provide theoretical reference and practical ideas for ultimately reducing the burden of disease.
